# Immune function analysis in a pediatric patient with a *de novo ALPK1* gene mutation

**DOI:** 10.3389/fimmu.2025.1622079

**Published:** 2025-10-29

**Authors:** Xue Chuan, Aiyan Ren, Shiyue Hu, Qian Li, Yin Zhu, Pingping Zhang, Lin Wang, Qing Du, Hong Li, Yan Chen, Pei Huang, Zuochen Du

**Affiliations:** ^1^ Department of Pediatrics, Affiliated Hospital of Zunyi Medical University, Zunyi, China; ^2^ Guizhou Children’s Hospital, Zunyi, China; ^3^ Department of Pediatric Surgery, Affiliated Hospital of Zunyi Medical University, Zunyi, China; ^4^ Department of Ophthalmology, Affiliated Hospital of Zunyi Medical University, Zunyi, China; ^5^ Collaborative Innovation Center for Tissue Injury Repair and Regenerative Medicine of Zunyi Medical University, Zunyi, China

**Keywords:** *ALPK1*, ROSAH syndrome, immune function, NF-κB pathway, targeted immunotherapy

## Abstract

**Introduction:**

ROSAH syndrome is a rare autosomal dominant disorder caused by heterozygous missense mutations in the *ALPK1* gene. It is clinically characterized by a spectrum of manifestations, including retinal dystrophy, optic disc edema, splenomegaly, anhidrosis, and headaches. This study performed an integrated evaluation of clinical manifestations, genetic alterations, and immunological profiles in a pediatric ROSAH syndrome case harboring an *ALPK1* mutation, with the objective of dissecting its putative immune-mediated pathogenesis.

**Methods:**

A 12-year-old male with unexplained splenomegaly and multisystem symptoms underwent clinical evaluation. Whole-exome and Sanger sequencing were used to identify *ALPK1* variants. Western blotting was applied to assess the activation status of the NF-κB pathway in peripheral blood mononuclear cells (PBMCs), coupled with flow cytometric characterization of T- and B-lymphocyte subset distributions. Clinical manifestations and treatment of ROSAH Syndrome caused by *ALPK1* mutations were summarized by literature review.

**Results:**

The individual presented with progressive visual loss, anhidrosis, migraine, and arthralgia. A heterozygous *de novo ALPK1* variant (c.710C>T, p.Thr237Met) was identified. Elevated phosphorylated IKKβ levels indicated NF-κB pathway activation. Lymphocyte profiling demonstrated markedly diminished CD3^+^ and CD8^+^ T-cell counts, with the CD4^+^/CD8^+^ ratio escalating to 2.61, concurrently with elevated proportions of activated CD4^+^, CD8^+^ T cells, and regulatory T-cell populations. B-cell lineage anomalies featured expansions in transitional B-cell subsets and plasmablasts, paralleled by reduced serum immunoglobulin concentrations. Approximately 70 cases have been reported globally, linked to four *ALPK1* mutations (c.710C>T, c.761A>G, c.830C>T, c.476C>T). Key clinical features include severe ocular involvement (retinal degeneration, optic nerve edema) and systemic manifestations such as splenomegaly and anhidrosis. Current management focuses on anti-inflammatory therapy and symptomatic support, while retinal degeneration remains untreatable.

**Discussion:**

This case links a *de novo ALPK1* mutation to constitutive NF-κB activation and immune dysfunction in ROSAH syndrome. Early genetic and immunological screening is essential for diagnosis and management of ROSAH syndrome. Targeted immunotherapy may alleviate symptoms, but further research is needed to define standardized approaches for this rare disorder.

## Introduction

Autoinflammatory diseases (AIDs) are complex multisystem disorders stemming from genetic mutations or acquired innate immune anomalies, triggering systemic inflammation via dysregulated immunological pathways. These diseases manifest as recurrent or persistent systemic inflammation, accompanied by marked elevation of acute-phase response proteins such as C-reactive protein and serum amyloid A, while lacking specific adaptive immune markers including autoreactive T lymphocytes or pathogenic autoantibodies (1). AIDs predominantly manifest during the neonatal period and early childhood, with epidemiological studies indicating that approximately 80% of cases experience initial onset before the age of 5. The hallmark clinical presentation involves episodic fever (persisting for days to weeks) accompanied by systemic inflammatory cascades and characteristic multisystem involvement, which may encompass cutaneous, musculoskeletal, respiratory, hematopoietic, neurological, gastrointestinal, ocular, and auditory systems (2). AIDs affect over 10% of the global population (3). These chronic conditions are difficult to treat, worsen quality of life over time, and significantly increase the risk of complications affecting multiple organs. Monogenic autoinflammatory diseases are classified based on dysregulated inflammatory pathways, including disorders driven by abnormal IL-1 activation (such as inflammasome dysfunction), extending to conditions with overactive type I interferon signaling, syndromes tied to NF-κB/TNF pathway imbalances, and subtypes caused by molecular mechanisms that remain incompletely understood (4, 5). As a central regulatory pathway of cellular activation, dysregulation of NF-κB activity underlies a spectrum of autoinflammatory and autoimmune disorders, including Blau syndrome, A20 haploinsufficiency, Deficiency of adenosine deaminase 2 (DADA2), Tumor necrosis factor receptor-1-associated periodic syndrome (TRAPS), and gain-of-function defects in Alpha-protein kinase 1 (ALPK1) (ROSAH syndrome) (6).

ROSAH syndrome is an autoinflammatory disorder characterized by childhood-onset splenomegaly and retinal dystrophy. Initial manifestations may include intermittent fever and incidentally detected pancytopenia (7). ALPK1 functions as a non-canonical α-kinase that exists primarily in a quiescent state within mammalian cellular systems (8). Operating as an intracellular pattern recognition sensor for microbial ADP-heptose (9). ALPK1 enzymatically modifies the forkhead-associated structural module of TIFA scaffolding protein through its catalytic kinase region. This enzymatic modification initiates TIFA oligomerization and subsequent signalosome formation, culminating in the activation of pivotal transcriptional regulators, NF-κB (nuclear factor kappa-light-chain-enhancer of activated B cells) (10) and AP-1 (activator protein 1) (11). These molecular events ultimately drive pro-inflammatory mediator production. Genetic mutations in *ALPK1* alter its substrate specificity for nucleotide diphosphate heptose (NDP-HEP), leading to constitutive kinase activation. This pathological activation sustains TIFA phosphorylation and thereby activates TIFA-dependent signaling cascades, even without microbial infection (11). The sustained signaling promotes continuous production of pro-inflammatory mediators, ultimately leading to chronic inflammatory pathogenesis.

In this study, we comprehensively evaluated the clinical, and immunological profiles of a 12-year-old boy carrying a *de novo ALPK1* mutation and presenting with manifestations consistent with ROSAH syndrome. We combined genetic analysis, protein expression studies, and immunophenotyping to investigate the molecular and immunological consequences of this variant. In addition, we reviewed the clinical manifestations and therapeutic strategies of ROSAH syndrome reported in the literature. Through these evaluations, our study aims to deepen the understanding of the mechanisms by which *ALPK1* mutations disrupt immune homeostasis and to provide insights for improving early diagnosis and precision management of ROSAH syndrome.

## Methods

### Patient

This study enrolled a 12-year-old male patient and age- and sex-matched healthy control subjects. Patient information was obtained following written authorization from the statutory custodians. The investigational framework was conducted in full compliance with ethical standards established by the Declaration of Helsinki. Formal ethics approval was granted by the Institutional Review Board of Affiliated Hospital of Zunyi Medical University.

### Genetic analysis

Genetic material was isolated from PBMCs of the patient-parent trio using the QIAamp DNA Mini Kit, following standard protocols. Whole-exome sequencing was conducted via Illumina NovaSeq 6000 at Connexus Medical Laboratory (Beijing). *ALPK1* variants were validated through Sange sequencing with custom primers: Forward: 5′-TAAGCAACAATGGAGCAACG-3′, Reverse: 5′-AAACACGTGCCACGGATATT-3′.

### Flow cytometry

Cellular immunophenotyping was performed to quantify peripheral lymphocyte subpopulations. PBMCs from the patient and age-matched healthy control were extracted and subsequently incubated with fluorophore-conjugated monoclonal antibodies, including: PerCP-anti human-CD3 (300326), FITC-anti human-CD4 (300506), Brilliant Violet 510™-anti human-CD8a (301048), APC/Cy7-anti human-CD31 (303120), PE-anti human-CD127 (IL-7Rα) (351304), PE/Cy7-anti human-CD45RA (304126), Brilliant Violet 421™-anti human-CD185 (CXCR5) (306920), Pacific Blue™- anti human-CD38 (356628), Brilliant Violet 605™- anti human-CD27 (302830), PE/Fire 640™- anti human-CD196 (CCR6) (353449), APC- anti human-CD183 (CXCR3) (353708), Brilliant Violet 711™- anti human-CD45RO (304236), Brilliant Violet 785™- anti human-PD-1 (329930), PE/Dazzle™ 594- anti human-CD57 (359620), AF660- anti human-TCRγ/δ (331240), Brilliant Violet 650™- anti human-CD25 (302634), APC/Fire™ 810- anti human-HLA-DR (307674), PerCP- anti human-CD38 (303520), AF488- anti human-CD24 (311108), APC- anti human-CD19 (302212), Brilliant Violet 510™- anti human-IgD (348220), PE- anti human-CD21 (354904), PE/Cy7- anti human-CD27 (302838), Brilliant Violet 421™- anti human-IgM (314516), APC/Cy7- anti human-CD23 (338520), and Brilliant Violet 711™- anti human-IgG (414740). Cellular specimens underwent Cytek Aurora-based cytometric profiling, with subsequent computational analysis using FlowJo v10.8.1 (BD Biosciences). Tabular results were generated through quantitative comparison with age-matched healthy controls.

### Western blotting

Cellular protein isolation from PBMCs was performed using RIPA buffer supplemented with protease inhibitor cocktail. Lysate aliquots were mixed with Laemmli buffer, separated on 10% SDS-PAGE gels, and transferred to PVDF membranes via electroblotting. After blocking with 5% non-fat milk, membranes were incubated with primary antibodies including anti-ALPK1 (19107-1-AP, Proteintech), anti-GAPDH (T0004, Affinity Biosciences), and anti-phospho-IKKβ (#2697, Cell Signaling Technology). Unbound antibodies were removed by three washes with TBST buffer. Species-matched HRP-conjugated secondary antibodies (RGAR001, 60004—1-Ig, Proteintech) were then applied. Following a final TBST wash, membranes were incubated with chemiluminescent substrate (premixed 1:1), and signals were captured using an enhanced chemiluminescence system (Bio-Rad).

### Summary of clinical manifestations and treatment of ROSAH syndrome

We systematically reviewed and synthesized the available literature on *ALPK1* mutations to delineate the clinical spectrum and therapeutic strategies for ROSAH syndrome.

## Results

### Case presentation

**Figure 1:**
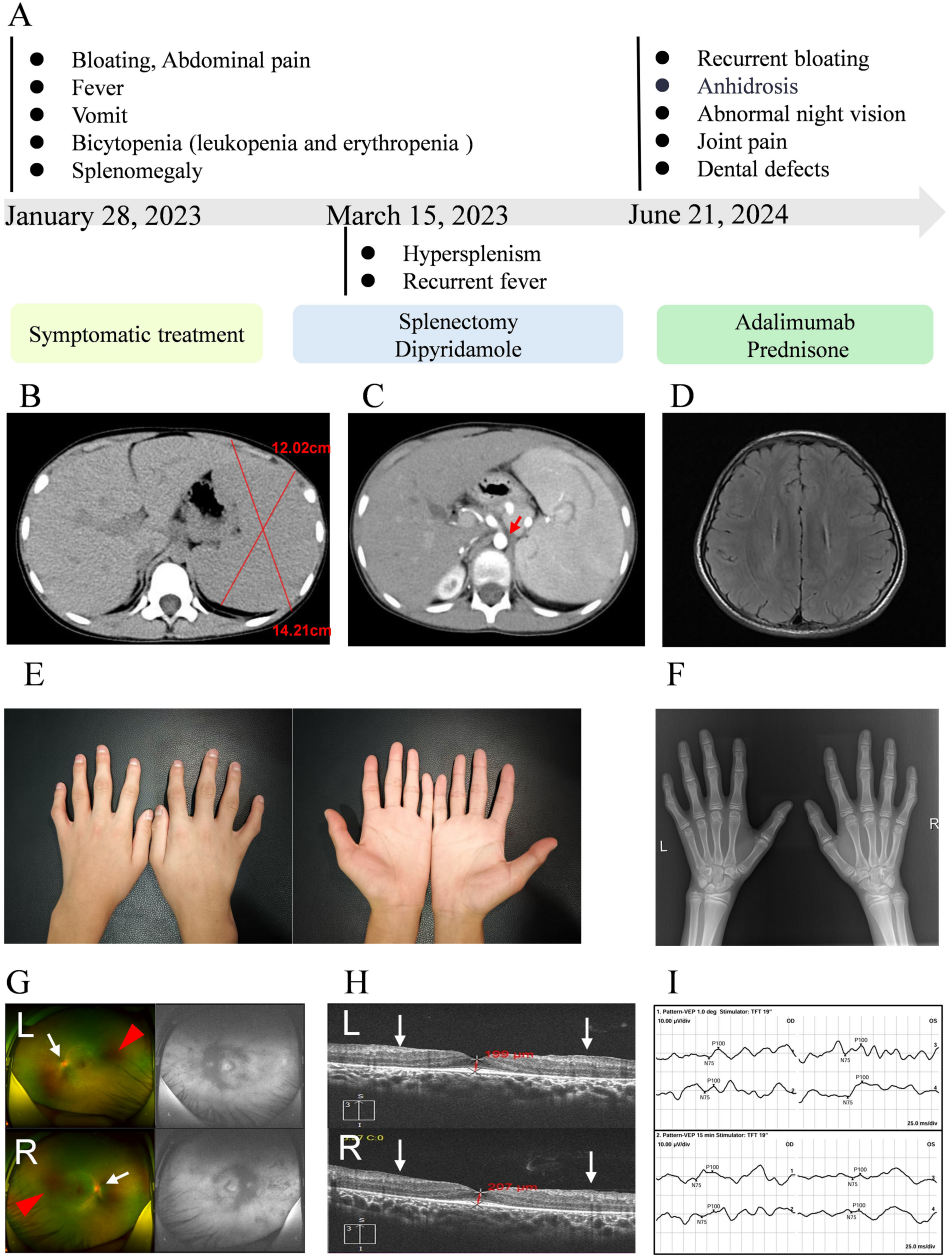
Clinical progression and phenotypic features. **(A)** Disease trajectory and temporal evolution. **(B, C)** Cross-sectional abdominal imaging; red arrows indicate dilation of the celiac trunk. **(D)** Cranial MRI. **(E)** Photographs of both hands. **(F)** Radiograph of the hands. **(G)** Fundus photographs (upper: left eye, lower: right eye); white arrows indicate blurred optic disc margins, red arrows indicate extensive hypofluorescent areas around the optic disc and nasal periphery. **(H)** Optical coherence tomography (OCT; upper: left eye; lower: right eye), white arrows indicate structural retinal abnormalities. **(I)** Visual evoked potential (VEP) testing (upper: 1.0-degree pattern VEP; lower: 15-minute pattern VEP).

This research documents the clinical progression of a 12-year-old male patient, as shown in **Figure 1A**. The patient had a history of postprandial vomiting and abdominal distension in infancy and early childhood, but no definitive diagnosis was made, and the family therefore did not pay significant attention. No family history of similar conditions was recorded. The patient was initially hospitalized in February 2023 with recurrent low-grade fever, vomiting, and paroxysmal abdominal pain. Imaging studies revealed splenomegaly and dilation of the celiac trunk (**Figures 1B, C**), accompanied by bicytopenia affecting the granulocytic and erythroid lineages. To correct the anemia, laparoscopic splenectomy was performed in March 2023. Postoperatively, hematologic parameters including C-reactive protein (CPR) levels normalized, and the patient was placed on regular follow-up.

At the 18-month postoperative follow-up, the patient reported intermittent headaches. Cranial magnetic resonance imaging (MRI) was performed and revealed no abnormalities of the brain (**Figure 1D**). Clinical examination revealed dental caries, which the patient had self-extracted, as well as symptoms of dry mouth. Color Doppler ultrasound of the salivary glands showed no significant abnormalities. In addition, mechanical stress–induced arthralgia of both hands and mild deformities of the interphalangeal joints were observed (**Figure 1E**). Radiographic and ultrasonographic assessments of the hand joints showed no abnormalities within the joint cavities (**Figure 1F**). During school age, the patient developed nyctalopia and progressive constriction of visual field, which showed partial improvement following vitamin A supplementation. After the definitive diagnosis of ROSAH syndrome was established, comprehensive ophthalmic examinations were performed to assess ocular involvement. Fundus photography revealed retinal pigmentary abnormalities, blurred optic disc margins, and a diminished foveal reflex (**Figure 1G**). Optical coherence tomography showed retinal thickening, an irregular inner retinal surface, and disorganization of retinal layers (**Figure 1H**). Visual evoked potential (VEP) testing demonstrated significantly prolonged P100 wave latency (right eye: 124 ms; left eye: 127.1 ms; reference range: 96.0–109.0 ms) and markedly reduced N75-P100 amplitude (right eye: 2.51 μV; left eye: 0.44 μV; reference range: 7.00–34.10 μV), indicating severe impairment of optic nerve conduction (**Figure 1I**). Taken together, these ophthalmic findings were consistent with severe dysfunction of the outer retinal layer. In addition, serum cytokine analysis revealed elevated levels of the inflammatory marker IL-10 (6.17 pg/mL; reference range <5.0 pg/mL).

After exclusion of infectious etiologies, low-to-moderate-dose prednisone combined with biologic therapy (adalimumab) was initiated to alleviate arthralgia, and achieved symptomatic remission prior to discharge. The patient was maintained on oral prednisone with scheduled subcutaneous adalimumab injections during follow-up. Concurrent symptoms were managed with individualized supportive care. All therapeutic interventions administered during the clinical course are summarized in **Table 1**.

**Table 1:** Summary of the clinical manifestations of the patient.

Involved systems	Manifestation	Clinical and laboratory findings	Treatment	Outcome
Ophthalmology	Progressive Vision Loss	Retinopathy	Adalimumab	Remission
Hematologic	Bicytopenia	WBC1.6x10^9^/L↓ HB 101g/L↓	Splenectomy	Cure
Digestive system	Abdominal pain Vomiting	Splenomegaly Enlarged Celiac Artery	Splenectomy	Cure
Dentistry	Dental caries	NA	Orthodontic Treatment	Remission
Neural system	Headache	(-)	Supportive	Remission
Immunology	Joint pain Fever	ANA (-), RF (-), Anti-CCP (-), Anti-GBM (-) IgG 18.9g/L↑ IgA 4.87g/L↑	Adalimumab	Remission

WBC, white blood cell; HB, Hemoglobin; ANA, antinuclear antibody; RF, rheumatoid factor; anti-CCP, Anti-Cyclic Citrullinated Peptide Antibodies; anti-GBM antibody, anti-glomerular basement membrane antibody; (-), Negative; NA, Not Available; ↑, Increase; ↓, Decrease.

**Figure 2:**
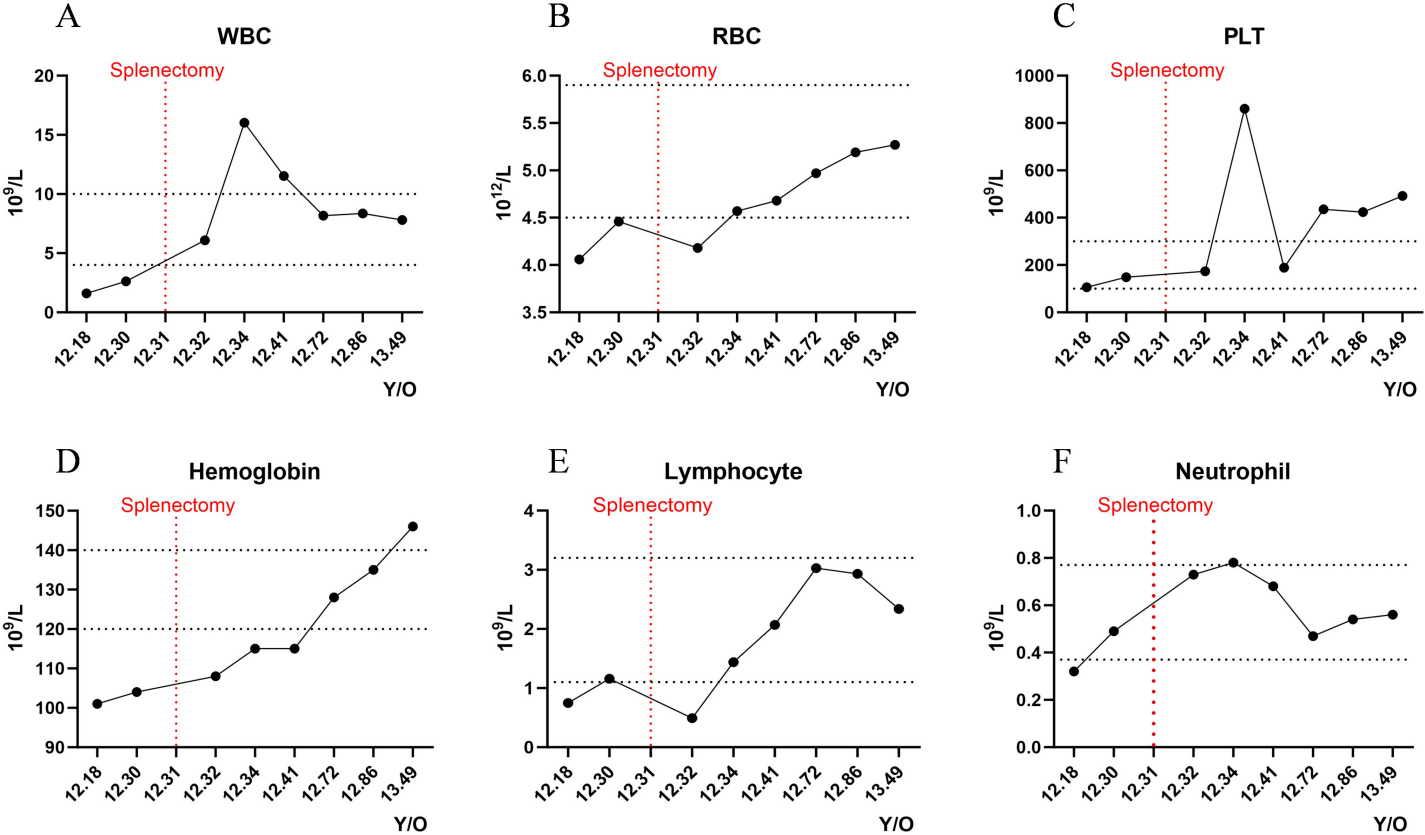
Longitudinal hematological monitoring. Dynamic fluctuations of key hematologic parameters during the disease course: **(A)** White blood cell count (WBC); **(B)** Red blood cell count (RBC); **(C)** Platelet count (PLT); **(D)** Hemoglobin (Hgb); **(E)** Lymphocyte count (Lymph); **(F)** Neutrophil count (Neut). Y/O, Years old.

Retrospective analysis of disease progression revealed initial bicytopenia, predominantly characterized by reductions in both leukocytes and erythrocytes. Following splenectomy, the patient's laboratory parameters, including white blood cell count, red blood cell count, neutrophil, and lymphocyte levels, gradually returned to normal, while hemoglobin showed a continuous upward trend. However, platelet counts increased above the normal range, prompting the initiation of dipyridamole therapy for thromboprophylaxis (**Figure 2**).

### Characterization of the *ALPK1 de novo* variant

**Figure 3:**
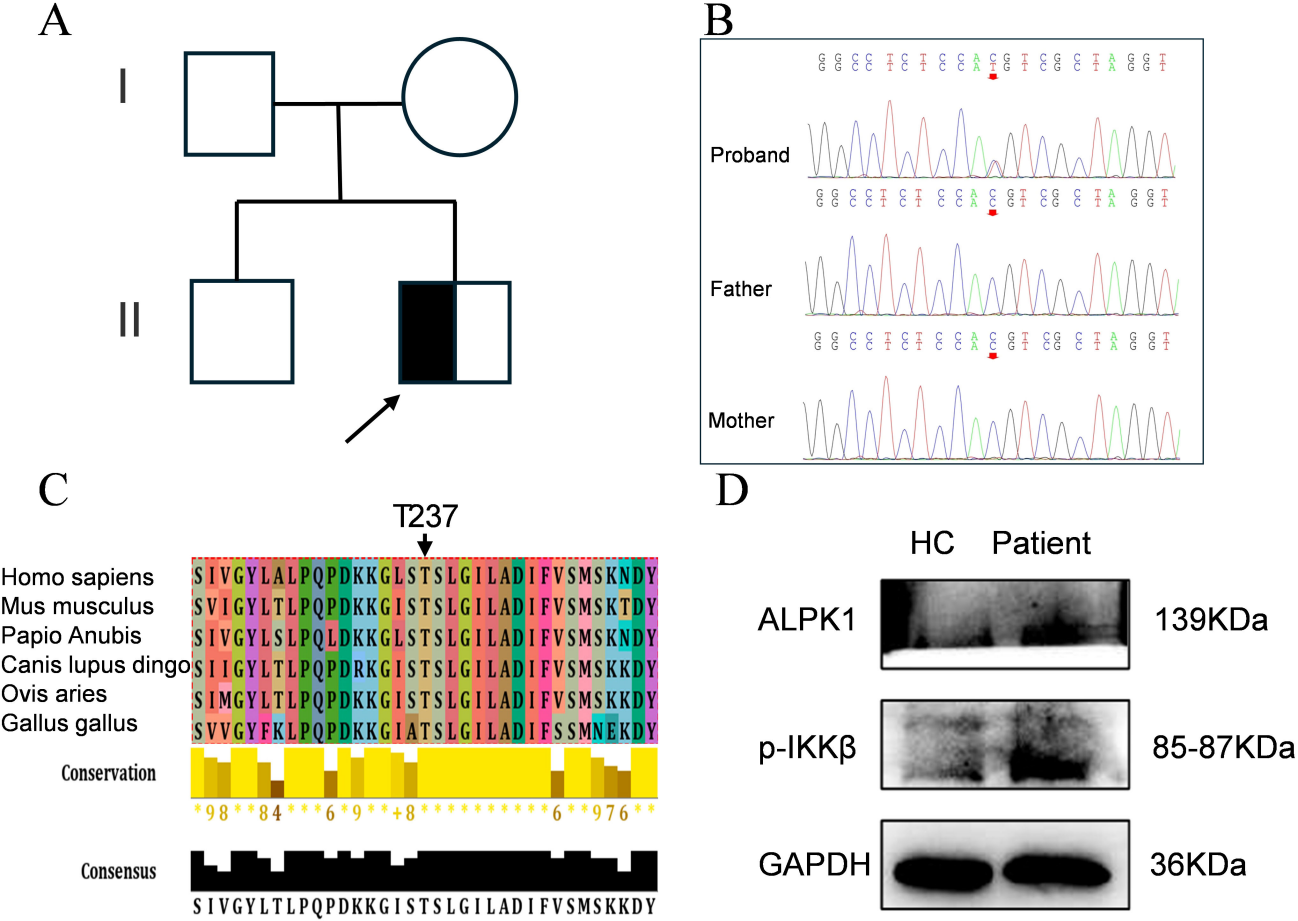
Identification of a *de novo* heterozygous missense mutation in *ALPK1*. **(A)** Family pedigree (males: squares; females: circles). **(B)** Sanger sequencing of the *ALPK1* in the patient and family members. **(C)** Evolutionary conservation of ALPK1 domains; conserved residues indicated by arrows. **(D)** Expression of ALPK1 and phosphorylated IKKβ in patient PBMCs and age-matched healthy controls (HC); GAPDH served as a reference.

As the case presented with progressive manifestations of hypersplhenism, anhidrosis, ophthalmic dysfunction, odontogenic abnormalities, and arthralgia, indicating pan-systemic pathology, we hypothesized that genetic factors might contribute to the disease pathogenesis. Whole-exome sequencing analysis of the proband and asymptomatic parental controls (**Figure 3A**) revealed a *de novo* heterozygous *ALPK1* variant (NM_025144: *c.*710C>T, p.Thr237Met) (**Figure 3B**), consistent with the clinical phenotype of ROSAH syndrome. Multiple sequence alignment analysis demonstrated high conservation of the affected amino acid residue (**Figure 3C**). Pathogenicity predictions using multiple bioinformatics tools uniformly supported its pathogenicity (**Table 2**).

**Table 2:** Prediction of ALPK1 mutation pathogenicity.

Variant	Provean score	Provean prediction	SIFT prediction	Mutationtaster prediction	PolyPhen-2 Hum-Div	PolyPhen-2 Hum-Var	ACMG rating
T237M	-2.43	Neutral	Damaging	Disease causing	1.0	0.94	Disease

Provean, Protein Variation Effect Analyzer; SIFT, Sorting Intolerant From Tolerant; PolyPhen-2 HumDiv, Polymorphism Phenotyping version 2 - Human Diverse; PolyPhen-2 Hum-Var, Polymorphism Phenotyping version 2 - Human Variation; ACMG Rating, American College of Medical Genetics and Genomics Rating.

To elucidate the effects of the ALPK1 Thr237Met variant on protein expression, we performed Western blotting to assess ALPK1 levels in PBMCs from the proband and healthy controls. The results showed increased phosphorylated IKKβ levels in the patient compared with controls (**Figure 3D**), indicating that the *ALPK1* mutation enhances activation of the NF-κB signaling pathway and increases ALPK1 protein activity.

### ALPK1 T237M variant alters lymphocyte subset distribution

**Figure 4:**
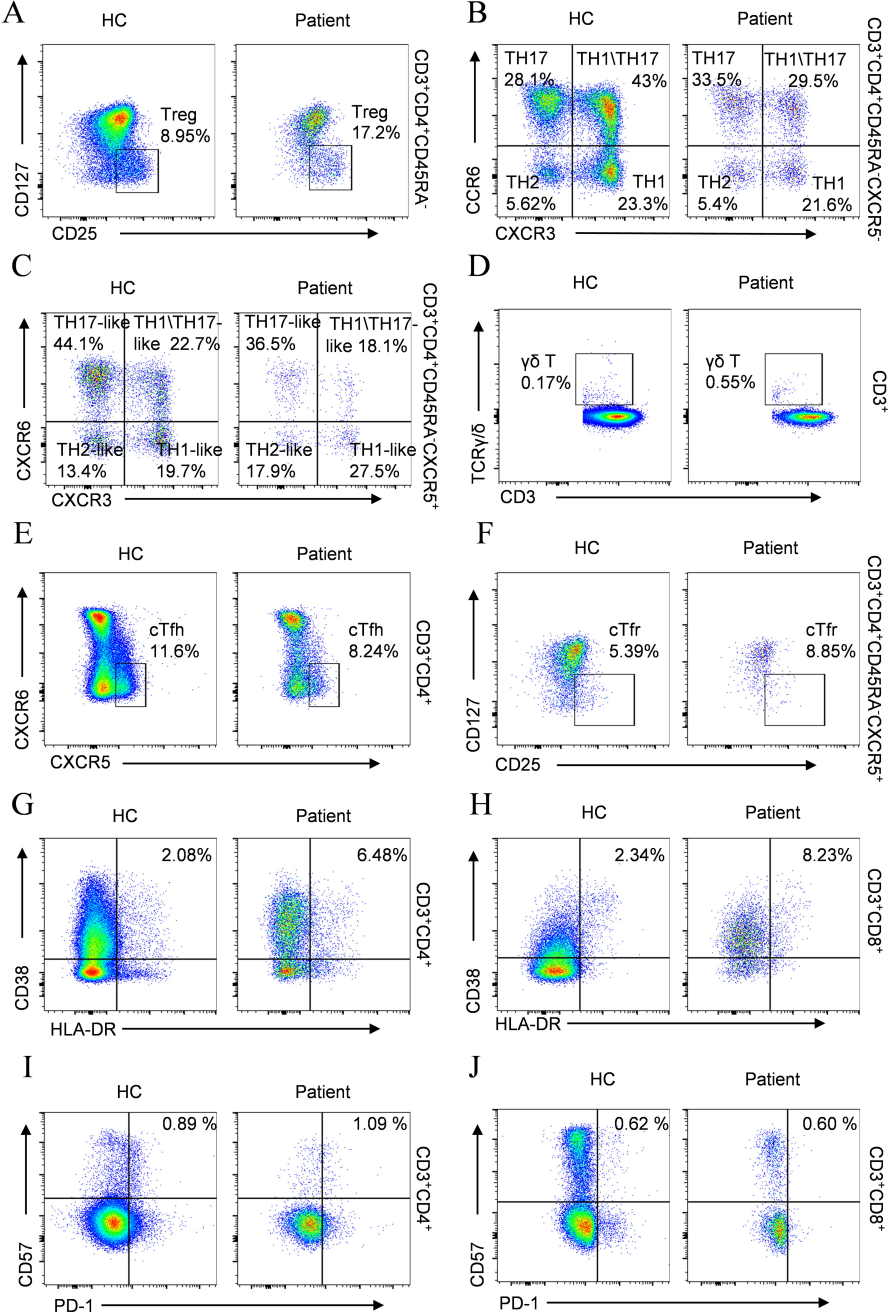
Comparative flow cytometry of T cell subsets in the patient versus age-matched HC. **(A)** Treg; **(B)** TH1/TH2/TH17; **(C)** cTfh subtypes (TH1/TH2/TH17-like); **(D)** γδ T cells; **(E)** cTfh; **(F)** cTfr; **(G)** Activated CD4^+^ T cells; **(H)** Activated CD8^+^ T cells; **(I)** Exhausted CD4^+^ T cells; **(J)** Exhausted CD8^+^ T cells.

Immunophenotypic profiling revealed marked alterations in lymphocyte subsets in the proband, with significant CD3^+^ and CD8^+^ T-cell lymphopenia observed compared with matched controls (**Table 3**). The CD8^+^ T-cell deficiency resulted in an inverted CD4^+^/CD8^+^ ratio, indicative of homeostatic disruption that may potentiate autoimmune pathogenesis. Further analysis of the CD4^+^ T-cell compartment revealed a marked expansion of regulatory T cells (Tregs) (**Figure 4A**). Immunophenotypic profiling revealed abnormalities in conventional Th1/Th2/Th17 lineages and their circulating follicular helper T-cell counterparts. Notably, classical Th17 cells were markedly expanded (**Figure 4B**), whereas Th17-like and Th1/Th17 populations were reduced (**Figures 4B, C**), reflecting a skewed inflammatory response with impaired follicular and hybrid effector functions.

**Table 3:** Immunological profile of the patient.

13 years				
Lymphocytes	Percentage	Reference range	Number/uL	Reference range
T cell	35.1(L%)	56.84-75.02	821.34	1184-2144
CD8^+^ T cell	8.69(L%)	21.91-36.80	203.35	489-1009
CD8^+^ naive	66.3(CD8%)	35.34-72.32	134.78	231-568
CD8^+^TEMRA	6.68(CD8%)	5.08-31.24	13.57	29-269
CD8^+^ CM	21.50(CD8%)	10.96-31.00	43.76	74-228
CD8^+^ EM	5.54(CD8%)	2.38-15.84	11.23	16-109
CD4^+^ T cell	22.7(L%)	22.25-39.00	531.18	522-1084
CD4^+^ naive	56.9(CD4%)	39.50-66.26	301.86	230-627
CD4^+^ TEMRA	1.33(CD4%)	0.00-1.54	7.02	0-12
CD4^+^ CM	32.6(CD4%)	25.34-49.90	173.39	182-403
CD4^+^ EM	9.16(CD4%)	4.68-15.70	48.67	29-117
CD4:CD8	2.61	0.65-1.65	NA	NA
B cell	15.9(L%)	8.84-17.76	372.06	203-476
Unswitched Memory B	17.9(B%)	7.15-23.10	66.69	20-86
Naive B	71.70(B%)	53.78-78.64	266.76	116-347
Transitional B	17.50(B%)	1.38-9.42	64.82	4-37
Plasmablast B	8.27(B%)	0.49-7.06	30.65	1-23.0

NA, Not Applicable. The red indicates an increase, while the blue signifies a decrease “L%” means “percentage of lymphocyte”. “CD8%” means “percentage of CD8 T cells”. “CD4%” means “percentage of CD4 T cells”. “T%” means “percentage of CD3 T cells”.”B%” means “percentage of CD19 B cells”.

Naïve: cytotoxic T lymphocyte with differentiation markers: CD3^+^ CD8^+^ CD45RA^+^ CD27^+^.

TEMRA: terminally differentiated effector memory: CD3^+^ CD8^+^ CD45RA^+^ CD27^−^.

CD8^+^ CM: central memory: CD3^+^ CD8^+^ CD45RA^−^ CD27^+^.

CD8^+^ EM: effector memory: CD3^+^ CD8^+^ CD45RA^−^ CD27^−^.

Naïve: helper T lymphocyte markers: CD3^+^ CD4^+^ CD45RA^+^ CD27^+^.

TEMRA: terminal effector memory differentiation: CD3^+^ CD4^+^ CD45RA^+^ CD27^−^.

CD4^+^ CM: central memory: CD3^+^ CD4^+^ CD45RA^−^ CD27^+^.

CD4^+^ EM: effector memory: CD3^+^ CD4^+^ CD45RA^−^ CD27^−^.

Unswitched Memory B cells, CD19^+^ CD27^+^ IgD^+^.

Naive B cells: CD19^+^ CD27^−^ IgD^+^.

Transitional B cells: CD19^+^ CD24^++^ CD38^++^.

Plasmablasts: CD19^+^ CD24^−^ CD38^++^.

Assessment of γδ T-cell populations revealed a marked increase in patients compared with healthy controls (**Figure 4D**). Aberrations in T follicular cell subsets were also observed, characterized by reduced cTfh cells (**Figure 4E**) accompanied by increased cTfr cells (**Figure 4F**). Analysis of T lymphocyte activation demonstrated elevated frequencies of activated CD4^+^ and CD8^+^ T-cell populations relative to healthy controls (**Figures 4G, H**). Concurrently, the frequency of exhausted CD4^+^ T lymphocytes was markedly increased (**Figure 4I**), whereas exhausted CD8^+^ T cells showed no significant difference (**Figure 4J**).

**Figure 5:**
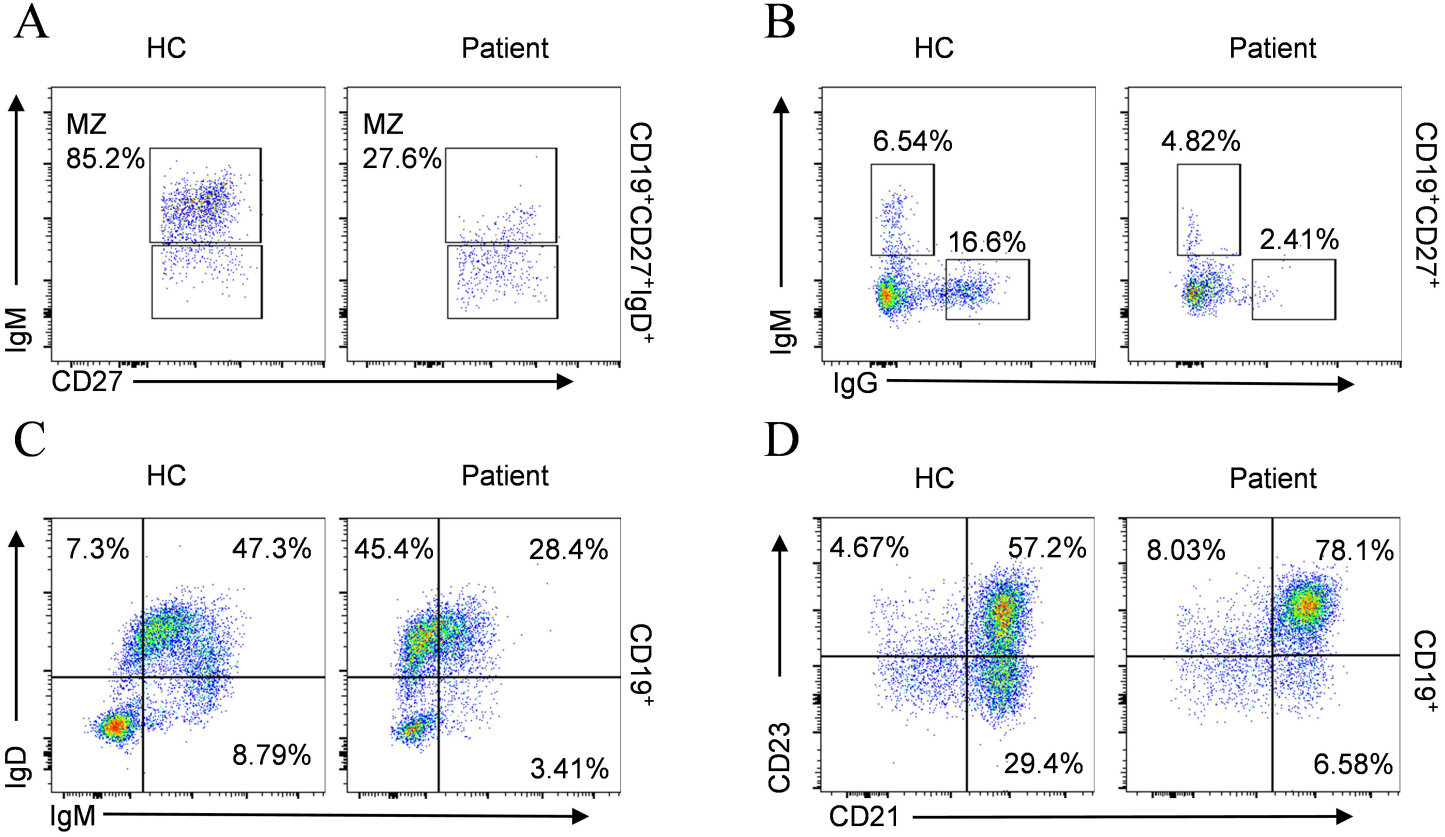
B cell phenotypic profiling compared with age-matched HC. **(A)** MZ B cells; **(B)** IgM^+^IgG^-^, IgM^-^IgG^+^ subsets; **(C)** IgD^+^IgM^-^, IgD^+^IgM^+^, IgD^-^IgM^+^ subsets; **(D)** CD23^+^CD21^-^, CD23^+^CD21^+^, CD23^-^CD21^+^ subsets.

Although the total B cell count remained within the normal range, both the percentage and absolute counts of transitional B cells and plasmablast were abnormally elevated (**Table 3**), suggesting aberrant activation in B cell differentiation. Comprehensive immunophenotyping of B-lymphocyte compartments further demonstrated marked depletion of marginal zone B cells (MZ B cells) (**Figure 5A**), together with disordered immunoglobulin expression patterns (**Figures 5B, C**) and an increase in CD23^+^CD21^+^ B cells accompanied by a decrease in CD23⁻CD21⁺ B cells (**Figure 5D**).

### Clinical manifestations and therapeutic management of ROSAH syndrome linked to *ALPK1* mutations: a comprehensive review

To date, approximately 70 cases of ROSAH syndrome have been reported worldwide. The currently identified pathogenic mutations include the high-frequency Thr237Met mutation, the Tyr254Cys mutation reported by Christina Torres Kozycki’s et al. (12), the Thr159Met mutation described by Song et al. (13), and the Ser277Phe mutation documented by Snelling et al. (14). Although these mutations share the core clinical triad, retinopathy, ectodermal dysplasia, and systemic inflammation, they exhibit distinct molecular mechanisms and phenotypic spectra (**Table 4**). Based on these findings, individualized treatment strategies can be implemented in clinical practice by tailoring interventions to patients’ specific symptoms.

**Table 4:** Summary of previously reported cases with ROSAH syndrome.

Mutations	Thr237Met	Tyr254Cys	Ser277Phe	Thr159Met
Mutation site	c.710C>T	c.761A>G	c.830C>T	c.476C>T
Location	N-terminal ADP-heptose binding domain	N-terminal ADP-heptose binding domain	N-terminal ADP-heptose binding domain	NA
ADP-heptose binding	Direct binding, loss of hydrogen bonds	Not directly combined	Indirect effects, loss of hydrogen bonds	NA
Human nucleotide sugar activation	UDP-mannose ADP-ribose	No activation	UDP-mannose ADP-ribose GDP-mannose	NA
Activation intensity	medium	Inactive	strong	NA
Kinase activity in the absence of ADP-heptose	Low or none	Unstable, inactive	high	NA
NF-κB/AP-1 activation	Dependent on human nucleotide sugars	Not activated	Dependent on human nucleotide sugars	NA
ADP-heptose dependence	Partially dependent, but can be activated by human nucleotide sugars	Not dependent at all	Partially dependent, but more easily activated by human nucleotide sugars	NA
Interactions with other amino acids	Affect ADP-heptose binding pockets	Affects Ser277 hydrogen bonding	Affects Ser277 hydrogen bonding and alters the specificity of ADP-heptose binding pockets	NA
Case report	62 cases	1 case	6 cases	1 case

NA, Not Available.

At present, ROSAH syndrome is primarily managed with immunosuppressants or biologic agents to control inflammatory progression, combined with symptom-directed supportive therapy (**Table 5**) and regular multisystem monitoring. Given the autosomal dominant inheritance pattern, genetic counseling is strongly recommended for probands and their first-degree relatives, as offspring have a 50% probability of inheriting the pathogenic allele. Follow-up data indicate that most patients experience alleviation of primary symptoms after standardized treatment (15). However, irreversible retinopathy currently remains without a curative intervention.

**Table 5:** Summary of clinical features and treatment approaches in ROSAH patients.

Manifestations	Frequency	Treatment
Optic nerve elevation	+++	Immunomodulation
Ocular inflammation	++	Immunomodulation
Splenomegaly	+++	Prophylactic splenectomy
Arthritis	++	Hormone therapy
Adiaphoresis	++	Symptomatic support
Headache	++	Symptomatic support
Enamel defects	++	Fluoride application
Episodic abdominal pain	+	Symptomatic support
Recurrent fever	++	Symptomatic support
Transient hemocytopenia	+	Symptomatic support

+++, Always present; ++, Frequently present; +, Occasionally present.

## Discussion

ROSAH syndrome is a rare auto-inflammatory disorder caused by pathogenic mutations in the *ALPK1* gene, which result in constitutive activation of the ALPK1-TIFA-NF-κB signaling pathway. In this study, we report a 12-year-old male patient with a *de novo ALPK1* p.Thr237Met mutation who presented progressive visual impairment, anhidrosis, splenomegaly, and arthralgia, representing the multisystem involvement typical of this condition. Molecular analyses suggested sustained activation of the NF-κB signaling pathway, while immunophenotyping revealed marked dysregulation of both T and B lymphocyte subsets. To date, approximately 70 cases of ROSAH syndrome have been reported worldwide, most associated with one of four recurrent hotspot mutations in *ALPK1*, and exhibit overall consistent phenotypes, with some variability in clinical presentation. Current treatment strategies rely primarily on glucocorticoids, immunosuppressants, or biologic agents to control inflammation, but none are effective in preventing or reversing retinal degeneration.

According to published studies, single nucleotide polymorphisms (SNPs) or deletion mutations in the *ALPK1* gene are genetically linked to the pathogenesis of multiple disorders (16), including Recurrent Periodic Fever (17), ROSAH syndrome, Chronic Kidney Disease (18), Myocardial Infarction, Ischemic Stroke, Spiradenoma, and Adenocarcinoma (19). However, distinct mutation sites in the *ALPK1* gene result in divergent clinical phenotypes. Disease-associated ALPK1 variants are principally implicated in two human pathologies: Thr237Met and Tyr254Cys alterations are mechanistically connected to ROSAH syndrome, while the Val1092Ala substitution constitutes 45% of spiradenoma and 30% of spiradenocarcinoma occurrences within analyzed clinical cohorts (20). In addition to the previously reported ALPK1 Thr237Met mutation causing ROSAH syndrome, Tyr254Cys, Thr159Met, and Ser277Phe mutations have also been demonstrated to induce this syndrome. These mutations result in functional aberrations of the ALPK1 protein, leading to dysregulated activation of inflammatory pathways (12–14). Significantly, all three genetic variants are positioned within the N-terminal domain of ALPK1 containing the ADP-heptose-binding region; however, exclusively the Thr237 residue occupies the ADP-heptose-binding pocket. Existing evidence reveals that ALPK1 Thr237Met and Ser277Phe substitutions enable constitutive activation via endogenous nucleotide sugars independent of ADP-heptose, while the Tyr254Cys variant induces complete ablation of ALPK1 catalytic function (12, 14). Notably, the Ser277Phe mutation exhibits the strongest activation potency by human nucleotide sugars and is uniquely responsive to UDP-mannose, a feature absent in Thr237Met or Tyr254Cys variants (14). In contrast, the functional impact of the ALPK1 Thr159Met mutation remains poorly characterized (**Table 5**).

The initial presentation of ROSAH syndrome is heterogeneous. Some patients first seek medical attention for abdominal pain, with subsequent evaluation revealing splenomegaly, as in the present case, whereas others present with recurrent unexplained fever (13) or progressive vision loss (21, 22), the latter often accompanied by irreversible retinal damage despite symptomatic treatment. Consistent with previously reported ALPK1 mutation–associated autoinflammatory disorders, our patient with the ALPK1 Thr237Met mutation exhibited the core manifestations of splenomegaly, vision loss, anhidrosis, and headache, together with additional features such as recurrent fever, arthralgia, and dental enamel hypoplasia (23). Notably, although he reported bilateral hand joint pain and mild deformity, imaging and serological evaluations did not support the presence of typical rheumatic disease. Dental caries was also observed, whereas salivary gland imaging was unremarkable. Neurological assessment, including brain MRI, was normal despite a history of headaches. In contrast, ophthalmic examination identified characteristic retinal abnormalities, which remain the most distinctive and diagnostically informative feature of ROSAH syndrome. Given its rarity and the limited understanding of its pathophysiology, misdiagnosis and underdiagnosis are frequent, leading to delayed management and irreversible end-organ injury. These findings underscore the importance of maintaining a high index of suspicion for hereditary autoinflammatory conditions in patients with unexplained multisystem involvement.

The relationship between systemic inflammation and cytopenia in ROSAH syndrome has been well established. Kozycki et al (12) systematically outlined the inflammatory spectrum of ROSAH syndrome, noting that patients often present with recurrent self-limiting low-grade fever and intermittent elevation of CRP. These inflammatory phenomena can occur in the absence of clear infection or other clinical symptoms and are frequently associated with hypersplenism and concurrent cytopenia (24). The underlying mechanism involves abnormal activation of the NF-κB and interferon signaling pathways, indicating that fever and CRP dynamics can serve as important indicators of the degree of inflammatory activity in the disease (12). In this case, non-infectious low-grade fever was also observed in the early stage of the patient, and the timing of fever coincided with the phase of hypersplenism-induced cytopenia. After splenectomy, along with gradual recovery of blood counts, the child’s body temperature and inflammatory markers also returned to normal. This outcome further supports the notion that fever and elevated CRP collectively reflect the underlying immune hyperactivation state in ROSAH syndrome and are associated with the mechanism of hypersplenism-mediated cytopenia (12). Therefore, dynamic monitoring of fever and CRP levels has important clinical significance for assessing disease activity and treatment response in ROSAH syndrome.

In addition to the previously reported neutrophil dysfunction in ALPK1-mutated patients and its association with disease activity (12, 24), our study highlights neutropenia as a notable manifestation. Interestingly, neutrophil counts partially recovered after splenectomy, suggesting a dual contribution of intrinsic cellular defects and extrinsic organ involvement to the hematologic phenotype of ROSAH syndrome. Immunophenotypic analysis conducted by Hecker et al. (25) revealed that the patient exhibited a reduction in CD8^+^ T cell numbers along with diminished production of IFN-γ and TNF-α. Although the potential influence of tofacitinib treatment cannot be entirely excluded, this agent only mildly inhibits cytokine secretion *in vitro*, suggesting that the observed reduction in cytokines is more likely related to the intrinsic disease mechanisms of ROSAH syndrome rather than pharmacological effects. Similarly, analysis of this pediatric patient showed decreased CD8^+^ T cell levels and an elevated CD4^+^/CD8^+^ ratio, indicating immune hyperactivation and impaired tolerance (26, 27). Naïve CD4^+^ T cells can differentiate into various subsets (28), and in this case, an expansion of Tregs was observed, possibly representing a compensatory mechanism to suppress excessive immune responses, though functional abnormalities may also be present (29). The expansion of γδ T cells suggests an amplified innate-like inflammatory response, which may synergize with Th17 dysregulation to exacerbate systemic immune imbalance (30). Concurrently, the patient exhibited elevated Th17 levels; this imbalance may synergistically promote inflammatory progression (31, 32). In addition to T cell abnormalities, previous literature (25) reported severe depletion of CD19^+^ B cells, largely attributable to prior rituximab therapy. Whether *ALPK1* mutation directly disrupts B cell homeostasis remains unclear. Although the total B cell count in this pediatric patient was within the normal range, the increased proportions of transitional B cells and plasmablasts exceeded physiological thresholds, suggesting dysregulated negative selection and a predisposition to autoimmunity (33). A reduction in marginal zone B (MZ B) cells was also observed, potentially associated with splenectomy, which may impair IgM synthesis (34, 35). During the germinal center (GC) reaction, impaired function of T follicular helper (Tfh) cells and dysregulated activity of T follicular regulatory (Tfr) cells may further affect B cell differentiation, maturation, and antibody class-switching (36, 37). Collectively, reduced immunoglobulin levels, diminished CD21 expression on B cells, and T cell deficiencies contribute to increased susceptibility to infections.

In the management of ROSAH syndrome, personalized treatment is critically important. At present, there are no effective therapies to alter the disease course or achieve complete remission. Clinical management primarily relies on anti-cytokine agents or biologics. Although these medications can effectively suppress progressive inflammation, their ability to prevent ocular damage remains limited (22, 23). Therefore, early involvement of ophthalmologists is imperative to develop more targeted treatment strategies for managing ocular complications. For systemic symptoms, active symptomatic management and rigorous monitoring of complications such as infections are essential to ensure timely intervention and minimize irreversible damage. DF-003, an orally bioavailable and highly selective ALPK1 inhibitor, targets the pathogenic ALPK1 T237M variant (IC_50_ = 16 nM) (38). Preclinical studies have demonstrated its ability to significantly suppress inflammatory cytokine expression and attenuate neuroglial activation in both the retina and central nervous system in a murine model of ROSAH syndrome (38). The compound has now advanced to clinical trials (NCT06395285), suggesting promising therapeutic potential in alleviating both ocular and systemic manifestations of the disease.

## Conclusion

In summary, detailed immunophenotyping results reveal abnormalities in T- and B-lymphocyte subsets in patients with ROSAH syndrome, which may contribute to immune dysfunction and multi-system involvement. Thus, early diagnosis and timely intervention are critically important. Lymphocyte subset analysis and comprehensive immunophenotyping during clinical evaluation not only facilitate monitoring of immune function changes and assessment of disease severity but also guide personalized treatment strategies, alleviate symptoms, and improve prognosis. Moreover, fluctuations in lymphocyte subset counts may serve as indicators of recovery status during disease remission. Given the rarity of this disorder, its precise immunoregulatory mechanisms and optimal therapeutic approaches warrant further investigation. Future studies should prioritize larger patient cohorts and robust statistical analyses to advance both basic and clinical research on ROSAH syndrome.

## Data Availability

The original contributions presented in the study are included in the article/supplementary material. Further inquiries can be directed to the corresponding authors.
